# Facile Fabrication of Cu_2_O Nanobelts in Ethanol on Nanoporous Cu and Their Photodegradation of Methyl Orange

**DOI:** 10.3390/ma11030446

**Published:** 2018-03-19

**Authors:** Zhenhua Dan, Yulin Yang, Fengxiang Qin, Hao Wang, Hui Chang

**Affiliations:** 1 Tech Institute for Advanced Materials and College of Materials Science and Engineering, Nanjing Tech University, Nanjing 210009, China; dan9506@gmail.com (Y.Y.); ch2006@njtech.edu.cn (H.C.); 2 The Synergetic Innovation Center for Advanced Materials, Nanjing Tech University, Nanjing 210009, China; 3 School of Materials Science and Engineering, Nanjing University of Science and Technology, Nanjing 210094, China; 4 Institute for Materials Research, Tohoku University, Sendai 9808577, Japan; hao.wang@imr.tohoku.ac.jp

**Keywords:** Cu_2_O nanobelts, porous materials, Azo dye degradation, photocatalysts, ethanol

## Abstract

Thin cupric oxide (Cu_2_O) nanobelts with width of few tens of nanometers to few hundreds of nanometers were fabricated in anhydrous ethanol on nanoporous copper templates that was prepared via dealloying amorphous Ti_40_Cu_60_ ribbons in hydrofluoric acid solutions at 348 K. The Cu_2_O octahedral particles preferentially form in the water, and nanobelts readily undergo the growth along the lengthwise and widthwise in the anhydrous ethanol. The ethanol molecules serve as stabilizing or capping reagents, and play a key role of the formation of two-dimensional Cu_2_O nanobelts. Cu atoms at weak sites (i.e., twin boundary) on the nanoporous Cu ligaments are ionized to form Cu^2+^ cations, and then react with OH^−^ to form Cu_2_O and H_2_O. The two-dimensional growth of Cu_2_O nanostructure is preferred in anhydrous ethanol due to the suppression of random growth of Cu_2_O nanoarchitectures by ethanol. Cu_2_O nanobelts have superior photodegradation performance of methyl orange, three times higher than nanoporous Cu.

## 1. Introduction

Massive dye-stuff is world-wide produced during the wet processing of textiles in the textile industry (i.e., annual more than 700,000 tons in 1980′s) [1]. Direct discharge of untreated dyes into the seas or rivers causes the serious environmental pollution, especially in less developed and developing countries [2]. Photochemical treatments of dye-containing effluent readily degrade dye molecules into CO_2_ and H_2_O, with no production of sludge and great reducing of foul odours [3]. Bulk cupric oxide (Cu_2_O) has a direct band gap of 2.17 eV, which makes it a promising material for the conversion of the solar energy into electrical or chemical energy [4]. Cu_2_O nanoarchitectures with different morphology, such as nanowires, nanocubes, and nanobars, are apt to contribute the superior catalytic performance for water splitting, solar energy conversion, and azo dye degradation due to the presence of Cu vacancies, which form an acceptor level 0.4 eV above the valence band [5]. Single crystalline Cu_2_O might be possible to convert photons into excitation with little loss to scattering, diffraction, and excitation could be converted back into photons to fulfill the cyclic application in solid state photovoltaic cells for the conversion of the solar energy [4,6,7]. On the base of superior photoactive properties of Cu_2_O, many efforts have been devoted to further improve their photochemical performance through chemi-physical manupilation of the shapes and dimensions of Cu_2_O. Cu_2_O nanostructures have been prepared by several different methods, such as the sonochemical method [8], thermal relaxation [9], liquid-phase reduction [10], the complex precursor surfactant-assisted route [11,12], and vacuum evaporation [13]. Cu_2_O can be also electrodeposited on several substrates, including Pt, Au, Cu, ITO, and stainless steel by the reduction of metal ions in suitable electrolyte solutions [14,15,16,17]. Recently, Cu/Cu_2_O-layered nanostructure materials with interesting optoelectronic properties have been prepared by step-wise electrodeposition [18,19]. The nanostructured Cu_2_O has a band gap of 2.61~2.69 eV, higher than that of bulk Cu_2_O [5]. The core-shell Cu@Cu_2_O nanocomposites formed on nanoporous Cu substrates via surface oxidation, as photocatalysts, have been reported to have an excellent photocatalytic activity and a good ability to degrade methyl orange pollutant with a factor of more than 20 [20]. Therefore, the Cu_2_O with a better nanostructure is of importance for the enhancement of its performances in azo dye degradation. The nanoporous copper as a substrate could increase the surface-to-weight ratio drastically, which is good for enhancing the optoelectronic performances. The nanoporous copper has been reported to enhance the surface enhanced Raman scattering effects by a factor of 1.85 × 10^5^ [21]. Therefore, Cu_2_O/nanoporous Cu could be a good candidate for azo dye degradation. The fabrication of Cu_2_O nanostructure on nanoporous Cu is of interest. The growth of Cu_2_O nanoarchitectures is affected by the stabilizing agents, such as polyvinylpyrrolidone [8,22]. In our former research, ethanol molecules are able to affect the two-dimensional growth of the Cu_2_O nanostructure on the nanoporous Cu templates with different morphology and defect densities [23]. However, the suppression mechanism of growth by ethanol molecule is needed to be investigated more in detail to clarify the effect of water molecule and ethanol in anhydrous ethanol. The enhancement of the azo-dye degradation by Cu_2_O nanostructure with different shapes is highly expected.

A facile fabrication of belt-shaped Cu_2_O nanostructures on nanoporous Cu templates in anhydrous ethanol is aimed in the present study. The formation mechanism of Cu_2_O nanobelts was discussed on the basis of the growth of Cu_2_O nanobelts in ethanol from SEM, TEM, and XPS analysis, and the degradation of methyl orange pollutant under the excitation of the daylight lamp source is evaluated.

## 2. Experimental Procedure

Ti_60_Cu_40_ ingots were made by an arc melting equipment (GSHL-500A, Haostar Materials Preparation Technology Co., Ltd., Shenyang, China). The ribbons were melt spun by a single-roller melt spinning equipment (XC-500, Shenyang Kejing Auto-instrument Co., Ltd., Shenyang, China). Nanoporous Cu (NPC) templates were prepared by dealloying these melt-spun Ti_40_Cu_60_ ribbons, with a width of 2 mm and thickness of ~25 μm in 0.03 M HF solution for 10.8 ks in HF-resistant glass bottles set in the water bath at 348 K. During dealloying, the bottles were turned up and down every 1200–1800 s. The dealloyed samples were washed by water for five times and then washed again by anhydrous ethanol for three times. NPC templates were then immersed into purchased chemical reagent anhydrous ethanol (purity >99.5 mass %, Sendai Wako Chemicals Co., Ltd., Sendai, Japan) for 24, 48, and 72 h. The water concentration of the anhydrous ethanol was about 0.5 mass %. The surface morphology of NPC templates was characterized by using scanning electron microscope (SEM, JEOL, FIB4610, JEOL Ltd., Tokyo, Japan). The crystalline states and the chemical compounds of reaction products on as-spun ribbons, dealloyed ribbons, and NPC templates after immersion in anhydrous ethanol were confirmed by X-ray diffractometor (XRD, Rigaku, RINT-4200, Rigaku Co., Tokyo, Japan). In order to figure out the effect of the ethanol on the formation of Cu_2_O nanobelts, the surface morphology of NPC after immersion in water (18 MΩ·cm at 298 K) for 72 h were also conducted. The microstructure of dealloyed ribbons and the Cu_2_O nanobelts was observed by a transmission electron microscope (TEM, JEOL, HC2100, JEOL Ltd., Tokyo, Japan) and a high-resolution transmission electron microscope (HRTEM, JEOL, ARM200, JEOL Ltd., Tokyo, Japan). The cross-sectional TEM and HRTEM samples were prepared by focused ion beam milling (FIB, JEOL, Dual beam FIB 4610, JEOL Ltd., Tokyo, Japan). Other TEM samples were prepared by the ion milling method.

Azo dye methyl orange (chemical formula: C_14_H_14_N_3_SO_3_Na), hereafter referred as MO, was degraded for 150 min under the excitation of the daylight lamp that was equipped with a ring-shaped LED lamp tube with a power of 40 W. The irradiation of the daylight lamp was conducted in the dark chamber covered by the black curtains. Four 100 mL glass tubes containing 50 mL MO test solution were set in the test-tube rack and tilted with a tilt angle of 30°. The ring-shaped LED tube of the daylight lamp, as an irradiation source, was set right above the rack. The light can cover all of the glass tubes readily because of the large diameter of ring-shaped LED lamp tube. The test glassy tubes with caps were set in the shaker with a speed of 280 rpm for 10 min in the dark to achieve adsorption equilibrium. Then, the suspension was exposed to the LED daylight lamp for 150 min. MO solution had an initial concentration of 20 mg L^−1^. These of belt-shaped Cu_2_O and NPC templates were 8, 10, 12 mg L^−1^, respectively. In order to compare the effect of Cu_2_O on the MO degradation, NPC with a loading amount of 10 mg L^−1^ was also tested. The total loading amount of octahedral Cu_2_O and NPC templates, which were treated in water for 24, 48, and 72 h, was 10 mg L^−1^. Scanning UV-Vis spectrometer (UV-Vis, Thermo-Fisher, Thermo Evolution 220, Thermo-Fisher Ltd., Waltham, MA, USA) with a wavelength of 465 nm was employed to analyze the degradation amount and efficiency of MO. The individual UV-Vis measurement was complemented by the baseline of the water calibrated every time for MO degradation experiments. The blank experiments show that the absorbance of the water solvent at the wavelength of 350–650 nm was confirmed to less than 0.001, lower than 0.06% of that of MO solution. The photodegradation ratio (*D*) was calculated according to the following equation: *D* = (*C*_0_ − *C*)/*C*_0_ × 100%, where *C*_0_ is the original absorbance of MO at its maximum absorbance wavelength and *C* is the absorbance of MO at the same wavelength after photodegradation. On the other hand, high Performance Liquid Chromatography (HPLC, SPD-20A, Shimadzu, Tokyo, Japan) was used to analyze the photodegradation performance of Cu_2_O@NPC on an analytical column C18 (4.6 mm × 250 mm, 5 μm) with elution of the water at a flow rate of 1.0 mL min^−1^. The column temperature was 35 °C. The change of the intensity of the voltage peaks can be used to reflect the degradation of MO.

## 3. Results and Discussion

### 3.1. Characteristics of Cu_2_O Nanobelts

**Figure 1:**
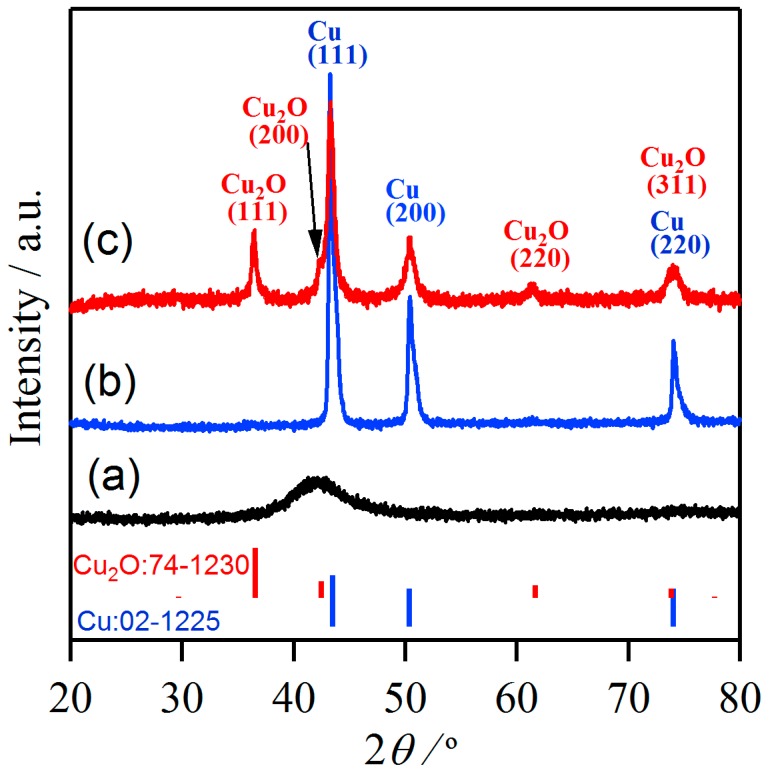
X-ray diffractometor (XRD) patterns of as-spun Ti_40_Cu_60_ ribbons (a), nanoporous Cu after dealloying in 0.03 M HF solution at 348 K (b) and nanoporous Cu after an immersion treatment in anhydrous ethanol for 72 h (c).

The crystalline state of surface oxides was confirmed by XRD. Only broad diffraction peak appeared in the XRD patterns of as-spun Ti_40_Cu_60_ ribbon (Figure 1a), indicating that the Ti_40_Cu_60_ ribbon had an amorphous structure. The blue and red vertical lines stand for the peak position and intensity of Cu (JCPDF card No.: 02-1225) and Cu_2_O (JCPDF card No.: 74-1230). As shown in Figure 1b, the diffraction peaks at 2Θ = 43.3°, 50.3°, 74.0° on the dealloyed ribbons after immersing in 0.03 M HF solution for 10.8 ks at 348 K were assigned to Cu (111), Cu (200), and Cu (220) in comparison to the standard *fcc* Cu patterns, indicating that the residue of dealloyed Ti_40_Cu_60_ ribbon was *fcc* Cu. After immersing the dealloyed samples in anhydrous ethanol for 72 h, the two main diffraction peaks centered at 2Θ = 36.5°, 61.5° were assigned to Cu_2_O (111) (220), and another diffraction peak at 42.3° from Cu_2_O (200) was overlapped by the diffraction peaks of *fcc* Cu around 2Θ = 43.3° in Figure 1c. The diffraction peaks from *fcc* Cu still exist. The presence of the diffraction peaks of *fcc* Cu at 2Θ = 43.3°, 50.3°, 74.0° may indicate that the Cu_2_O compound did not fully cover the NPC surface or the Cu_2_O nanostructures are too thin to prevent the X-ray penetration. The appearance of diffraction peaks from Cu_2_O compounds in Figure 1c demonstrated that the crystalline Cu_2_O formed after the free immersion of 72 h in anhydrous ethanol.

**Figure 2:**
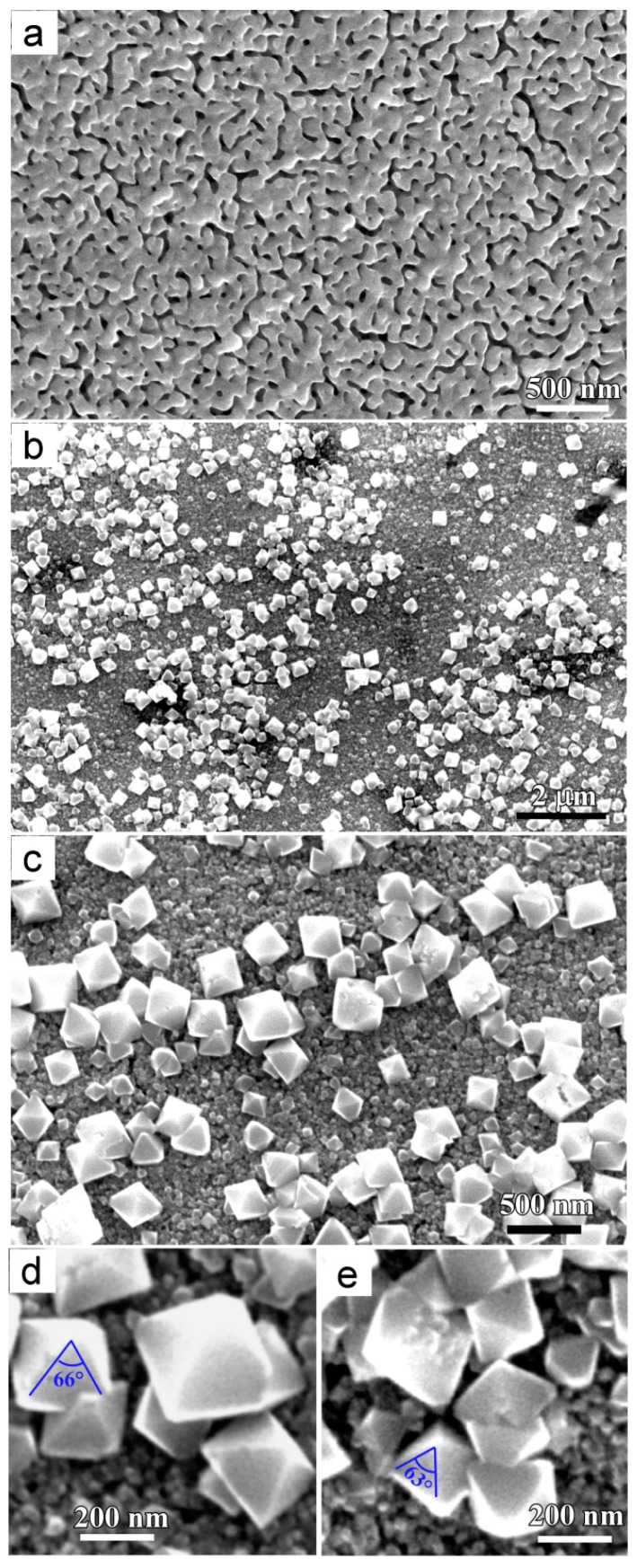
Microimages of nanoporous Cu before (**a**) and after an immersion treatment in water for 72 h (**b**), high magnified morphology (**c**) and octahedral Cu_2_O particles (**d**,**e**).

**Figure 3:**
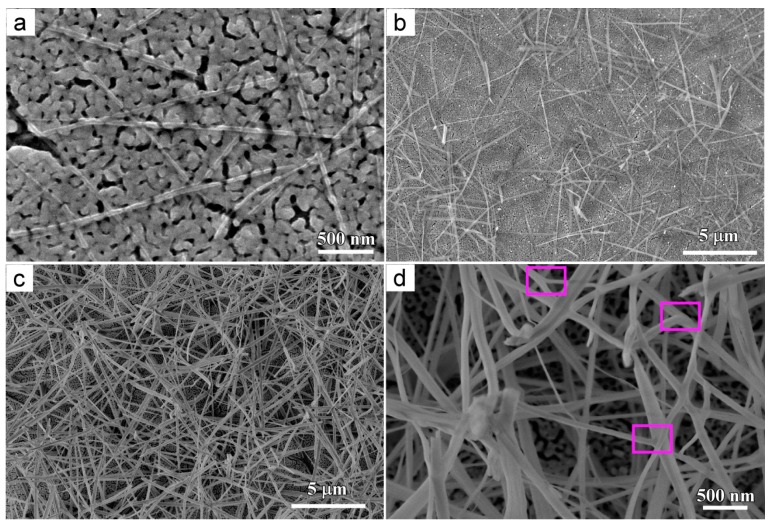
Microimages of Cu_2_O nanobelts on nanoporous Cu after free immersion in ethanol for 24 h (**a**), 48 h (**b**), 72 h (**c**), and high magnified morphology of c (**d**).

Figure 2 shows the morphology of NPCs before and after immersion in water. The uniform nanoporous structure was formed after dealloying Ti_40_Cu_60_ ribbon in HF solution (Figure 2a). As shown in the SEM image in Figure 2b and magnified SEM image in Figure 2c, many octahedral particles formed on NPC samples after free immersion in water for 72 h. The high magnified particles in Figure 2d,e show that the octahedral particles had a vertex angle of 63° ± 5° and edge length of few tens of nanometers to few hundreds of nanometers. The atomic concentrations of the oxygen and copper were confirmed to be 69.1 at% and 30.9 at% by SEM-EDX analysis. The Cu/O ratio of the octahedral particles was close to 2. It is thus concluded that the chemical composition of particles are Cu_2_O. According to E–pH diagram of Cu in H_2_O at 298 K, the critical pH for the formation of Cu_2_O is higher than 6 [24]. Therefore, the formation of Cu_2_O phase is favorable for highly active NPC in water with pH 7. After immersing NPC in anhydrous ethanol for 24 h, several belt-shaped nanostructure, hereafter referred as nanobelt, formed on the surface of NPCs, as shown in Figure 3a. The nanobelts had a width of about 40 nm and few micrometers in length. The nanobelts had a thickness much smaller than their length and width since the nanobelts are transparent under secondary electron observation. After free immersion in anhydrous ethanol for 48 h, more Cu_2_O nanobelts formed, as shown in Figure 3b. The nanobelts had a width of about 162 nm. Many nanobelts grew in length more than 7 μm. After free immersion in anhydrous ethanol for 72 h, much more Cu_2_O nanobelts covered the NPC surface and most of them crossed with others. The mean width of the nanobelts was confirmed to be 166 nm, and many of them are several tenth micrometers long, as shown in Figure 3c. The nanoporous structure underneath the nanobelts looked similar with those in Figure 2a. It is worth noting that many small branches grew along the other Cu_2_O nanobelts in Figure 3d, marked in three frames. When compared with Figure 2 and Figure 3, it is considered that the immersion of NPC in anhydrous ethanol helped in forming the two-dimensional Cu_2_O nanobelts. On the other hand, the Cu_2_O nanobelts preferred the dominant growth in the two dimensions of width and length and the slow thickening. After immersion of 48 h in anhydrous ethanol, the width of Cu_2_O nanobelts almost remained constant. Cu_2_O nanobelts tended to become longer with the increase of the immersion time.

**Figure 4:**
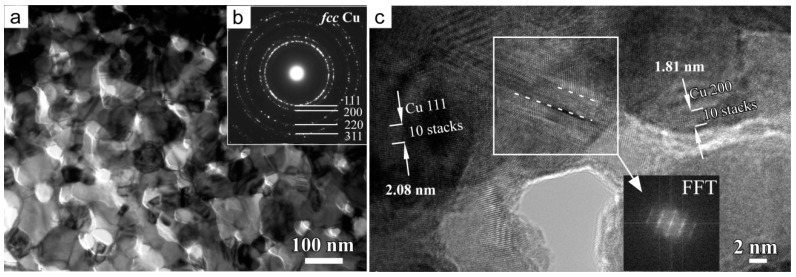
Cross-sectional bright field TEM image (BFI) (**a**), selected area diffraction pattern (SADP) (**b**), and high-resolution transmission electron microscope (HRTEM) image (**c**) of the internal part of nanoporous Cu substrate after free immersion in ethanol for 72 h. The dash lines in the frame marks the position of the interface of twin boundaries in the Cu ligaments and the corresponding fast Fourier transform (FFT) pattern is inserted in c.

The bright field TEM image (BFI) of the internal part of NPC templates after immersion of 72 h in ethanol demonstrated that NPCs had a mean pore size of 38 nm and a mean ligament size of 46 nm in Figure 4a (measured over more than 125 sites of internal part of NPC templates by Nanomeasure^®^ software). The diffraction rings in the selected area diffraction pattern (SADP) was assigned to *fcc* Cu, and there were no diffraction rings from Cu_2_O phase. The HRTEM image shows a crystalline phase existing in the ligaments. The interpanal distance between the adjacent fringers was confirmed to be about 2.08 nm and 1.81 nm for 10 stacks of the lattice panels, which corresponded to Cu (111) and Cu (200). On the basis of XRD (Figure 1b) and TEM data (Figure 3b), the NPC template was composed of *fcc* Cu. In addition, there are many twin boundaries existing in the crystalline Cu ligaments, as shown in high resolution bright field TEM image in Figure 4c. The fast Fourier transform (FFT) pattern was similar to the diffraction patterns typical of metastable phases, indicating the existence of the defects of the stacking faults of the twin boundaries in the white frame.

**Figure 5:**
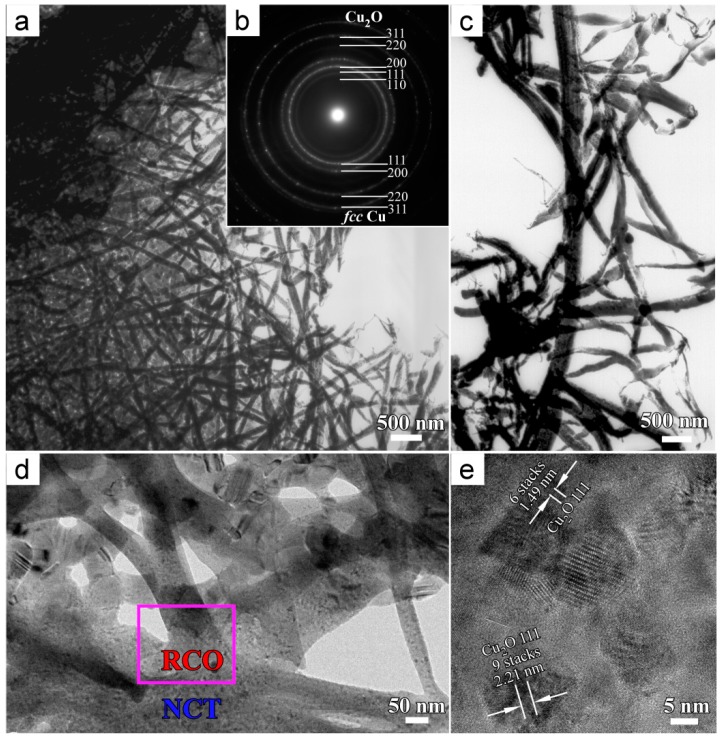
BFI (**a**,**c**), SADP (**b**), high-magnified cross-sectional transmission electron microscope (TEM) image (**d**) and corresponding HRTEM image (**e**) of Cu_2_O nanobelts formed on the surface of the nanoporous Cu after free immersion in ethanol for 72 h (RCO: Root of Cu_2_O nanobelts; NCT: Nanoporous copper templates).

The BFIs of NPC template in Figure 5a,c,d after immersion in anhydrous ethanol for 72 h show the similar morphology of SEM morphology in Figure 3c. Long nanobelts crisscrossed with others. The two inner diffraction rings from Cu_2_O (110) and (111), and one from Cu_2_O (220) clearly appeared in Figure 5b. The diffraction rings from *fcc* Cu were also observed because Cu_2_O formed on the surface of NPC templates. On the basis of XRD (Figure 1c) and TEM (Figure 5b) data, the nanobelts formed on NPC templates after immersion in anhydrous ethanol for 72 h were confirmed to be a nanocrystalline Cu_2_O phase. When compared with SADP of the internal part of NPCs, the appearance of the diffraction rings from Cu_2_O (110), (111), and (220) demonstrated that the amount of Cu_2_O nanobelts on NPC surface was much higher than that in the internal. As shown in Figure 5c, there were some parts in left lower corner where the nanobelts interlaced, and the long nanobelts formed in some parts. In fact, many branches grew along some wide nanobelts which are considered to form prior to other narrow nanobelts. To some extent, this fact demonstrated the two-dimensional (2D) growth process of Cu_2_O nanobelts in anhydrous ethanol. The BFI on the root of Cu_2_O nanobelts in the frame in Figure 5d shows that the Cu_2_O nanobelts grew up from the NPC ligaments. The interplanar distance between the fringers was confirmed to be 0.245 nm (2.21 nm for nine stacks and 1.49 nm for six stacks marked in Figure 5e), which corresponded to that of Cu_2_O (111). The HRTEM image on the root of the nanobelts proved that the nanobelts consisted of the crystalline Cu_2_O phase. This fact in Figure 5 shows that the Cu_2_O nanobelts preferentially nucleated at some specific sites (i.e., twin boundary, lamellar defects or dislocations) and then grew up in 2D model (i.e., Growth in length).

**Figure 6:**
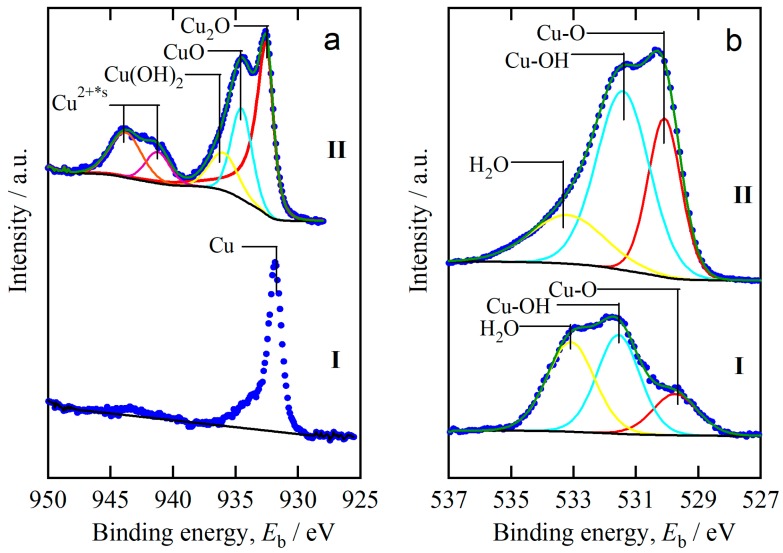
XPS spectra of Cu 2p (**a**) and O 1s (**b**) of nanoporous Cu before (**I**) and after (**II**) free immersion in anhydrous ethanol.

The chemical bonding state of the dealloyed samples before and after immersion in anhydrous ethanol is shown in Figure 6. As shown in Figure 6a-I, the peak at 931.9 eV corresponded to the metallic Cu in the dealloyed Ti_40_Cu_60_ ribbons. The Cu or Cu_2_O species that existed on the NPC surface were considered to be formed through oxidation reaction of oxygen in the air during transferring the samples since the hydrogen bubbles were generated during dealloying amorphous Ti_40_Cu_60_ ribbon. In addition, the absence of diffraction peaks in XRD pattern (Figure 1b) and the diffraction rings (Figure 4b) of Cu_2_O phase also proved that the formation of Cu_2_O species resulted from the oxidation reaction of oxygen in the air. Simultaneous interactions of outgoing photoelectrons with a valence electron excites to a higher-energy level accompanying with the presence of satellite peaks [25]. The presence of d^10^ Cu(I) spectra in Figure 6a-II indicates the formation of the Cu_2_O compound on the surface. As shown in Figure 6a-II, the shake-up sub-peaks at 934.6 and 936.2 eV were assigned to CuO and Cu(OH)_2_ compounds, respectively. The symbol, Cu^2+*s^, stands for the satellite peaks of Cu 2p. Simultaneous interactions of outgoing photoelectrons with a valence electron excites to a higher-energy level accompanying with the presence of satellite peaks [25]. The Cu 2p spectrum after immersing NPC templates in anhydrous ethanol for 72 h suggested that the Cu existed in the form of Cu_2_O, CuO and Cu(OH)_2_ compounds. When compared with the spectrum of Cu 2p after dealloying, the main difference is the appearance of the oxidation of Cu. The metallic Cu atoms were oxidized by the water molecules in anhydrous ethanol. The Cu_2_O compound was predicted as the main reaction products. The spectra of O 1s are shown in Figure 6b. The three kinds of bonding were confirmed to be M-O, M-OH, and absorbed water. Since the cation species were only Cu^2+^, the atomic concentration of Cu(OH)_2_ was 1.8 times higher than that of CuO. The CuO species were considered to result from the oxidation reaction of Cu_2_O in the air. Cu(OH)_2_ species were considered to be the intermediate reaction products of the formation of Cu_2_O. When compared with the XPS spectra before and after immersion in anhydrous ethanol, the concentration of Cu_2_O and other CuO, Cu(OH)_2_ species drastically increased due to the formation of Cu_2_O and the intermediate reaction products.

### 3.2. Formation Mechanism of Cu_2_O Nanobelts

During dealloying Cu_30_Mn_70_ alloy in 1 mM HCl solution at 0 V vs. Ag/AgCl reference electrode, the Cu_2_O nanocubes were formed at pH around 3 [21]. The Cu_2_O with different shapes (i.e., nanothreads, nanowires, nanocubes) was synthesized by anodic oxidation of Cu in pure water [5]. They proposed the formation process, as described by the following reactions in water:

2H_2_O → O_2_(O-O)_SA_ + 4H^+^ + 4e^−^ (1)

2Cu → 2Cu^2+^ + 4e^−^ (2)

4H^+^ + 4e^−^ → 2H_2_ (3)

2Cu^2+^ + (O-O)_SA_ + 4e^−^ → Cu_2_O + O_SA_ (4)

2Cu + 2H_2_O → 2H_2_ + Cu_2_O + [O]_SA_ (5)

where SA stands for surface absorbed species. A continuous layer of adsorbed oxygen is formed on the Cu anode. The Cu^2+^ ions generate from the oxidation of Cu electrode (Re. 2) and combine with adsorbed oxygen (O_SA_) to form Cu_2_O nanowires. At low pH, the reaction (Re. 2) is prevailed. However, at higher pH 8.5~10, the following reaction is favored [26]:

Cu^2+^ + 2OH^—^ → Cu_2_O + H_2_O (6)

Lou has reported that the addition of NaOH plays a crucial role in the formation of the flower-like Cu_2_O nanoarchitectures [27]. The dropwise of NaOH enhances the preferential growth of Cu_2_O along (110) planes and the continuous growth of Cu_2_O crystals helps in forming the thin nanopetals and nanoflowers [27]. The Cu_2_O compounds preferentially form when solution pH is between 8 and 10, and the alkaline solution enhances the reduction reaction of cupric (Cu^2+^) cations to cuprous (Cu^+^) cations, as described in Re. 6 [28]. In our experiments, chemical reagent of anhydrous ethanol contained a 0.5 mass % water. The formation of Cu_2_O nanobelts in ethanol is considered to have a similar formation mechanism as that in the alkaline solutions described above. As shown in Figure 3b, there were many defects (i.e., twin boundary, kinks at the edge of ligaments) in the NPC ligaments, which served as initial sites for the ionization. The Cu^2+^ ions involved in Re. 2 was formed from the ionization reaction of NPC substrate in weakly alkaline conditions. The formation of intermediate reaction products in Re. 6, Cu(OH)_2_, was confirmed by XPS analysis (Figure 5a). Meanwhile, the observation of the root of Cu_2_O nanobelts indicated that the nanobelts grew from the Cu ligaments, as shown in Figure 5d. In our case, Re. 3 does not prevail due to the abundant ethanol molecules. The water molecules react with dissolved O_2_ to generate OH^−^, then shift pH to a weakly alkaline, and further serve as a reservoir of OH^−^. Therefore, the formation of Cu_2_O nanobelts is considered to be followed reaction Re. 1, 2, 5, 6. The defects, i.e., twin boundary and kinks, as shown in Figure 4c, served as a seed for the formation of Cu_2_O. It is worth stating that no Cu_2_O nanobelts or nanocubes were formed on the inner ligaments after immersion of 72 h in ethanol, as indicated by Figure 4. It is reasonable to assume that the pH is different from site to site between inside NPCs and in the bulk ethanol due to the diffusion limits of water and ethanol molecules. Therefore, the formation of Cu_2_O nanobelts is caused by the geometrical properties of NPCs, local pH level, diffusion of ions, and water. Summarily, the formation of Cu_2_O nanobelts experienced several steps: (1) Dissolution: The generation of Cu^2+^ ions from the defects in the Cu ligaments; (2) Diffusion: The diffusions of ions, water, and ethanol through the nanopores into the bulk ethanol under the interaction of the concentration gradients; (3) Nucleation 1: The formation reaction of intermediate products, Cu(OH)_2_, between Cu^2+^ cations and OH^−^ or absorbed O_2_; (4) Nucleation 2: The reduction of Cu(OH)_2_ into Cu_2_O nanopetals; (5) Growth 1: The accumulation of Cu_2_O at the preformed Cu_2_O seeds at the defects on NPC ligaments; and, (6) Growth 2: The continuous growth of Cu_2_O along the (111) panels to form the nanobelts as indicated by the high diffraction intensity of Cu_2_O (111) at 36.6°. Although the accurate concentration of Cu_2_O nanobelts was not measured in the present study, the limited concentration of H_2_O in ethanol determined the rare formation of Cu_2_O nanobelts. In the whole process, the H_2_O molecules as reactants help in the formation of Cu_2_O nanobelts and ethanol molecule functionalized as a stabilizing or capping reagent for inhibiting the growth of metal nanoparticles [29,30,31]. It is believed that the highly localized reduction of the constrained ions might be responsible for the formation of the flat, highly anisotropic shape, like the nanobelt shape. It is therefore presumed that the introduction of ethanol molecules affects the diffusion process at the metal/electrolyte interface and 2D growth of nanobelts. As a result, the Cu_2_O molecules grew along the length direction to form the nanobelts. The slight change in the width of Cu_2_O nanobelts during the immersion in ethanol for 48 and 72 h is considered to be supportive evidences.

### 3.3. MO Photodegradation Performance of Cu_2_O Nanobelts

**Figure 7:**
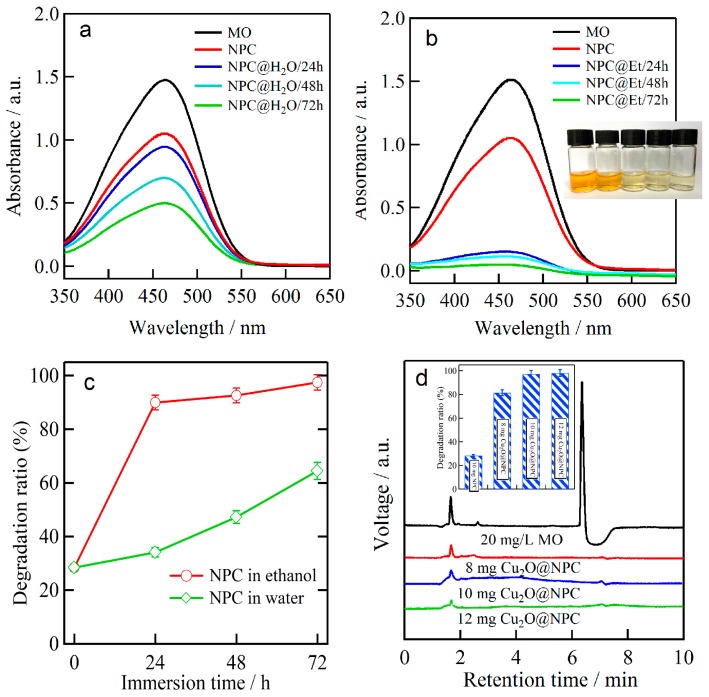
UV-Vis spectra of MO degradation of octahedral cupric oxide (Cu_2_O) and Nanoporous Cu (NPC) (**a**), Cu_2_O nanobelts and NPC (**b**) colour change of MO solution (inset in b), degradation amount of 20 mg/L MO photodegraded by Cu_2_O nanobelts and NPC and octahedral Cu_2_O and NPC for different times (**c**) and HPLC chromatograms of MO after photodegradation by Cu_2_O@NPC with load of 8, 10, and 12 mg L^−1^ (**d**). The inset bar plots in d are the photodegradation ratio of corresponding NPC and Cu_2_O@NPC from UV-Vis spectrometer. Et stands for ethanol. NPC@H_2_O stands for the NPC immersion in water; NPC@Et stands for the NPC immersion in ethanol, Cu_2_O @NPC stands for Cu_2_O nanobelts and NPC.

The photocatalytic performance of the Cu_2_O nanobelts was evaluated by the photodegradation of MO under daylight lamp irradiation for 150 min. As shown in Figure 7a, the UV-Vis absorption spectra demonstrate that the intensity of the characteristic absorbance peak of MO at its maximum absorbance wavelength (λ = 465 nm) decreases with the immersion time of NPCs in water. The octahedral Cu_2_O particles and NPC exhibited a weak ability for MO degradation, as shown in Figure 7a. The continuous increase of MO degradation ratio is considered to be due to the increase of Cu_2_O amount from the spontaneous formation reactions in the water with pH 7, with an increase of immersion time in water [24]. The degradation ratio of octahedral Cu_2_O particles and NPC after 150 min of irradiation of daylight lamp was measured to be 34.1% after immersion in water for 24 h, 47.2% after immersion in water for 48 h, and 64.4% after immersion of 72 h, respectively. The NPC without the distribution of Cu_2_O species had a degradation ratio of 28.4%, which is slightly lower than that of octahedral Cu_2_O particles and NPC after immersion in water for 24 h, and about two times lower than that of octahedral Cu_2_O particles and NPC after immersion in water for 72 h. When Cu_2_O nanobelts and NPC were used to degradation agents, much more MO molecules were oxidized and the absorbance was close to zero as shown in Figure 7b. The inset image shows the fade of the colour during photodegradation of MO with more load of Cu_2_O nanobelts. The degradation ratio of MO reached 88.9% for the NPC templates that were immersed in ethanol for 24 h, 92.6% for NPC templates immersed in ethanol for 48 h, and 97.4% for NPC templates immersed in ethanol for 72 h, as shown in Figure 7c. When compared with the MO degradation performance between octahedral Cu_2_O particles and Cu_2_O nanobelts, Cu_2_O nanobelts exhibit much better degradation efficiency. In addition, we also investigated the effect of the load amounts on the photocatalytic activity of the Cu_2_O nanobelts. HPLC chromatograms of MO after photodegradation by Cu_2_O@NPC with load of 8, 10, and 12 mg L^−1^ in Figure 7d shows that the residual MO concentration is very low by the drastic decrease of peaks at the retention time of 1.643 and 6.357 min in comparison to the initial MO pollutant solution. The drastic decrease of voltage peaks at 6.357 min demonstrates that Cu_2_O@NPC photocatalysts are effective and efficient to degrade MO in form of breaking the “-N=N-” azo bonding [32,33]. The degradation ratio of MO after irradiation of 150 min reached 30.6% for 10 mg L^−1^ bare NPC, 81.5% for 8 mg L^−1^, 97.4% for 10 mg L^−1^, and 98.1% for 12 mg L^−1^ loaded Cu_2_O nanobelts and the NPC mixture formed in ethanol for 72 h as shown in the inset bar plot in Figure 7d. When exposed to the daylight lamp, the p-type Cu_2_O semiconductor can be excited to produce electrons and holes. The photo-generated electrons and holes can trigger a series of photodegradation reactions. Holes at the valence bands readily oxidize hydroxyl ions (OH^—^) adsorbed on the surface of the Cu_2_O photocatalyst to yield hydroxyl radicals (OH) in Re. 5, which play an important role in MO photodegradation [34].·OH has a higher redox potential (+1.9 V) [35] than that of MO (+0.94 V) [36], which is why OH, rather than the photogenerated holes (+0.519 V), can degrade adsorbed MO [37]. On the other hand, the photo-generated electrons conducted away from holes by Cu can be captured by adsorbed oxygen molecules (O_2_) in Re. 4 and 5, leading to the generation of hydrogen peroxide (H_2_O_2_) and OH^−^, or super oxide radical ions (O_2_^−^) [38]. The O_2_^−^ ions can further interact with intermediate H_2_O_2_, which facilitates the yield of OH^−^ and OH and in return contributes to the photodegradation process of MO [37]. Finally, MO can be oxidized into intermediates and be desorbed from the surface of Cu_2_O. In the present case, only 28.4% MO was degraded by 10 mg L^−1^ NPC after 150 min of the irradiation of daylight lamp. However, more than 97% MO were degraded by the mixture of Cu_2_O nanobelts and NPC. The photodegradation of MO was increased about 69%, as indicated in Figure 7b,c, due to the efficient degradation of the involvement of Cu_2_O nanobelts on NPC formed in ethanol. As has been reported, the photodegradation of Cu_2_O nanoarchitectures obeys the pseudo-first-order rule (ln (*C*/*C*_0_) = K*t*) and the degradation ratio is linearly dependent on the degradation time [20]. On the basis of Figure 3, Figure 4 and Figure 5, Cu_2_O nanobelts grew along these NPC ligaments and covered partially NPC without high distribution of Cu_2_O inside the nanoporous structure. Therefore, small amount of Cu_2_O nanobelts is considered to form on NPC and functionalize as the agents for the photodegradation of MO. The degradation rates of Cu_2_O nanobelts@NPC agent in Table 1 [20,39,40,41] (0.68 mg min^−1^ g_cat_^−1^ for 8 mg L^−1^ for Cu_2_O nanobelts@NPC, 0.65 mg min^−1^ g_cat_^−1^ for 10 mg L^−1^ for Cu_2_O nanobelts@NPC and 0.55 mg min^−1^ g_cat_^−1^ for 12 mg L^−1^ for Cu_2_O nanobelts@NPC) were drastically higher than that of 10 mg L^−1^ NPC. The Cu_2_O nanobelts might exhibit better MO photodegradation performance due to the shape effect [22,42]. It is obviously that the Cu_2_O nanobelts have much better MO degradation performance than other Cu_2_O nanocubes or polyhedras [39,41]. Worse degradation rate of MO than core-shell Cu@Cu_2_O might be due to the difference in the mass of the degradation agents on Cu ligaments, since the wholly Cu_2_O-covered Cu ligaments with the large surface area should be abundant in the mass and surface area in comparison to the nanobelts. The Cu_2_O nanobelts mainly composed of (111) planes are able to enhance the adsorption of MO molecules on the exposed (111) surface, and serve as stable photocatalysts to further improve the photodegradation of azo dye, as indicated by the high intensity of Cu_2_O (111) in Figure 1 [22,34]. The MO dye molecules are adsorbed on the surface of the p-type Cu_2_O semiconductor nanobelts and act as electron donors. Meanwhile, the photo electrons are excited by the daylight source due to the narrow band gap (i.e., 2.17–2.69 eV) of Cu_2_O nanocrystals [4,5]. These injecting electrons from their excited states into the conduction band of the Cu_2_O nanobelts under daylight irradiation accelerate the production of superoxide and hydroxyl radicals, and drastically improve the reaction rates of MO photodegradation. The p-type Cu_2_O semiconductors are known to absorb a relatively large amount of oxygen as O_2_^—^ on the surface [43], which further accelerate the reaction rates of MO degradation as well. Wholly speaking, the belt-shaped Cu_2_O nanostructures have almost four times higher photodegradation rate than NPC, and one order higher than polyhedral Cu_2_O and other Cu_2_O microcrystals [42]. The photocatalytic efficiency of TiO_2_ and ZnO photocatalysts with a dose of 1 g L^−1^ for 25 mg L^−1^ methyl orange at pH 4 for 240 min has been reported to be about 40% and 70% under UV irradiation [44]. The photodegradation behaviour of TiO_2_ and ZnO catalysts follows the pseudo-first-order kinetic rules and is similar with Cu_2_O@NPC. However, the photodegradation efficiency of Cu_2_O@NPC is much higher than that reported value of TiO_2_ and ZnO. All of the facts above might be attributed to the high rereactivity and large surface area of Cu_2_O nanobelts. The present strategy for the fabrication of nanobelt-shaped Cu_2_O phase can be extended to other nanoporous metals, such as nickel, tin, or cobalt.

## 4. Conclusions

The Cu_2_O nanobelts with width of few tens of nanometers and a length of few tens of nanometers to several micrometers were facilely synthesized on nanoporous Cu templates after free immersion in anhydrous ethanol. The key point for the formation of the Cu_2_O nanobelts is that abundant stabilizing agent ethanol and small amount of water served as a reservoir of OH^−^ and reactants. Both of ethanol and water molecules preferred the 2D growth of Cu_2_O nanobelts. The Cu atoms on the defects such as twin boundaries or kinks at the edge of the Cu ligaments were ionized to generate Cu^2+^ ions, and then these Cu^2+^ ions reacted with OH^−^ in the ethanol to form Cu_2_O species. The formation of Cu_2_O nanobelts on nanoporous Cu template experienced four steps: (1) Dissolution of defects on nanoporous template; (2) Diffusion of ions through nanopores; (3) Nucleation of Cu_2_O; and, (4) 2D growth of Cu_2_O nanobelts. The Cu_2_O nanobelts with large reaction surface area exhibited superior degradation performance of methyl orange, three times higher than bare nanoporous copper due to their high surface area and photocatalytic ability of Cu_2_O {111} facet.

## Figures and Tables

**Table 1:** Comparison of degradation efficiency for Cu_2_O nanobelts@NPC, Core-shell Cu@Cu_2_O, Cu_2_O nanocubes, and Cu_2_O@Cu.

Photocatalyst	Additive Amount	MO Concentration	Degradation Ratio	Irradiation Time	Degradation Rate	Light Source
	mg	mg L^−1^	%	min	mg min^−1^ g_cat_^−1^	
Cu_2_O nanobelts@NPC (This work)	8	20	81.5	150	0.68	40 W daylight lamp (460–610 nm)
	10	20	97.4	150	0.65	
	12	20	98.1	150	0.55	
NPC (This work)	10	20	28.4	150	0.19	
Core-shell Cu@Cu_2_O [20]	4	20	90	100	2.25	Sun light (300–2500 nm)
Cu_2_O nanocube [39]	8	20	83.6	120	0.70	300 W xenon lamp (190–1100 nm)
Cu_2_O@Cu [40]	30	10	90	120	0.14	40 W tungsten lamp (350–2500 nm)
Cu_2_O polyhedral [41]	50	15	96	180	0.06	500 W xenon lamp (≥400 nm)
Cu_2_O octahedra [41]	50	15	80	180	0.05	500 W xenon lamp (≥400 nm)
